# Bioinformatics Identified 17 Immune Genes as Prognostic Biomarkers for Breast Cancer: Application Study Based on Artificial Intelligence Algorithms

**DOI:** 10.3389/fonc.2020.00330

**Published:** 2020-03-31

**Authors:** Zhiqiao Zhang, Jing Li, Tingshan He, Jianqiang Ding

**Affiliations:** Department of Infectious Diseases, Shunde Hospital, Southern Medical University, Shunde, China

**Keywords:** breast cancer, disease-free survival, immune gene, transcription factor, prognostic signature

## Abstract

An increasing body of evidence supports the association of immune genes with tumorigenesis and prognosis of breast cancer (BC). This research aims at exploring potential regulatory mechanisms and identifying immunogenic prognostic markers for BC, which were used to construct a prognostic signature for disease-free survival (DFS) of BC based on artificial intelligence algorithms. Differentially expressed immune genes were identified between normal tissues and tumor tissues. Univariate Cox regression identified potential prognostic immune genes. Thirty-four transcription factors and 34 immune genes were used to develop an immune regulatory network. The artificial intelligence survival prediction system was developed based on three artificial intelligence algorithms. Multivariate Cox analyses determined 17 immune genes (ADAMTS8, IFNG, XG, APOA5, SIAH2, C2CD2, STAR, CAMP, CDH19, NTSR1, PCDHA1, AMELX, FREM1, CLEC10A, CD1B, CD6, and LTA) as prognostic biomarkers for BC. A prognostic nomogram was constructed on these prognostic genes. Concordance indexes were 0.782, 0.734, and 0.735 for 1-, 3-, and 5- year DFS. The DFS in high-risk group was significantly worse than that in low-risk group. Artificial intelligence survival prediction system provided three individual mortality risk predictive curves based on three artificial intelligence algorithms. In conclusion, comprehensive bioinformatics identified 17 immune genes as potential prognostic biomarkers, which might be potential candidates of immunotherapy targets in BC patients. The current study depicted regulatory network between transcription factors and immune genes, which was helpful to deepen the understanding of immune regulatory mechanisms for BC cancer. Two artificial intelligence survival predictive systems are available at https://zhangzhiqiao7.shinyapps.io/Smart_Cancer_Survival_Predictive_System_16_BC_C1005/ and https://zhangzhiqiao8.shinyapps.io/Gene_Survival_Subgroup_Analysis_16_BC_C1005/. These novel artificial intelligence survival predictive systems will be helpful to improve individualized treatment decision-making.

## Introduction

As the most common malignant tumor in women, breast cancer (BC) resulted in 2,088,849 new cases and 626,679 deaths in 2018 (1). Although advances in diagnosis and treatments improved the survival rate of patients with early BC, but the survival rate of patients with advanced BC was still poor (2). Early identification of BC patients with poor prognosis and timely individualized treatments were helpful to improve the prognosis of BC patients.

The tremendous progress of bioinformatics has provided tremendous support for exploring the intrinsic mechanism of tumorigenesis and prognosis. (3–6). Tumor-infiltrating immune cells were reported to be associated with tumorigenesis and prognosis (7, 8). It was reported that there was a significant correlation relationship between tumor-infiltrating immune and prognosis in BC patients (9). Immune-related genes could be used to calculate the immune scores and evaluate the tumor infiltration of immune cells to analyze the tumor immune characteristics (10). There were several prognostic models for prediction of prognosis in BC patients (11–13). However, these prognostic models could only provide mortality risk prediction for patients in different subgroups, but not individual mortality risk prediction for a specific patient at the individual level. From a specific patient's point of view, the patient's own mortality risk prediction was more important than that of patients in different subgroups. Therefore, a prognostic model that can provide individualized mortality risk prediction for a specific patient is helpful to optimize individualized treatment and improve clinical prognosis.

The current research aimed at exploring the relationship of immune-related genes with transcription factor, immune-infiltrating cells, and disease-free survival (DFS) of BC patients. Based on different artificial intelligence algorithms, the current study focused on developing artificial intelligence survival predictive systems for providing individual mortality risk prediction for BC patients.

## Materials and Methods

### Study Datasets

The original gene expression dataset from The Cancer Genome Atlas (TCGA) database contained 21,205 mRNAs from 1,109 tumor specimens to 113 normal specimens. After removal of patients with survival time <1 month and duplicate samples, 1,030 were included in further survival analyses. The original gene expression values have been log_10_ transformed for TCGA dataset. GSE31448 dataset (GPL570 platform) contained 246 patients and 23,319 mRNAs.

Gencode.v29 was used for converting probe IDs name to gene symbols.

### Differentially Expressed Analyses

Differentially expressed analyses were conducted with cutoff values of log_2_ |fold change| >1 and *P* < 0.05 by “edgeR” (14). Data were normalized by Trimmed mean of M values method.

### Immune Gene and Transcription Factor

Immune genes were identified through Immunology Database and Analysis Portal database (15). Transcription factors were defined through Cistrome Cancer database (16).

### Tumor Immune Infiltration

Six tumor-infiltrating immune cell data were obtained from Tumor IMmune Estimation Resource database (16). Single sample gene set enrichment analysis was used to evaluate tumor immune infiltration scores for 28 immune categories (17, 18).

### Statistical Analyses

Statistical analyses were conducted by SPSS Statistics 19.0 (SPSS Inc., Chicago, IL, USA). Artificial intelligence algorithms were performed by Python language 3.7.2 and R software 3.5.2. Artificial intelligence algorithms were carried out according to the original articles: Cox survival regression (19), multitask logistic regression (20, 21), and random survival forest (22, 23). Threshold for statistically significant difference was *P* < 0.05.

## Results

### Study Datasets

Details of research steps are displayed in Supplementary Figure 1. Table 1 displays the basic information of patients in the model dataset and validation dataset. The mortality rate in the validation dataset was 32.1% (79/246), which was significantly higher than 19.6% (202/1,030) in the model dataset.

**Table 1:** Clinical features of included patients.

	**TCGA dataset**	**GSE31448 dataset**	***P***
Number [*n*]	1,030	246	
Death [*n* (%)]	202 (19.6)	79 (32.1)	<0.001
Total survival time (mean ± SD, month)	40.1 ± 35.8	61.1 ± 41.2	<0.001
Survival time for dead patients (month)	45.0 ± 37.4	37.7 ± 28.5	0.080
Survival time for living patients (month)	38.9 ± 35.3	72.1 ± 41.7	<0.001
Age (mean ± SD, year)	58.3 ± 13.2	55.2 ± 13.5	<0.001
AJCC PT (T3)	166 (16.1)	68 (27.6)	0.002
AJCC PT (T2)	588 (57.1)	121 (49.2)	
AJCC PT (T1)	274 (26.6)	57 (23.2)	
AJCC PT (NA)	2 (1.9)	0	
AJCC PN (N1)	529 (51.4)	129 (52.4)	0.662
AJCC PN (N0)	482 (46.8)	115 (46.7)	
AJCC PN (NA)	19 (1.8)	2 (0.8)	
AJCC PM (M2)	157 (15.2)	NA	
AJCC PM (M1)	20 (1.9)	NA	
AJCC PM (M0)	853 (82.8)	NA	
AJCC PM (NA)	0	NA	
Progesterone receptor (positive)	665 (64.6)	124 (50.4)	<0.001
Progesterone receptor (negative)	320 (31.1)	120 (48.8)	
Progesterone receptor (NA)	45 (4.4)	2 (0.8)	
Estrogen receptor (positive)	763 (74.1)	NA	
Estrogen receptor (negative)	224 (21.7)	NA	
Estrogen receptor (NA)	43 (4.2)	NA	
Grade 3	NA	116 (47.2)	
Grade 2	NA	84 (34.1)	
Grade 1	NA	44 (17.9)	
Grade 0	NA	3 (1.2)	

*Continuous variables are presented as mean ± standard deviation. NA, missing data; AJCC, American Joint Committee on Cancer*.

### Differentially Expressed Analyses

**Figure 1:**
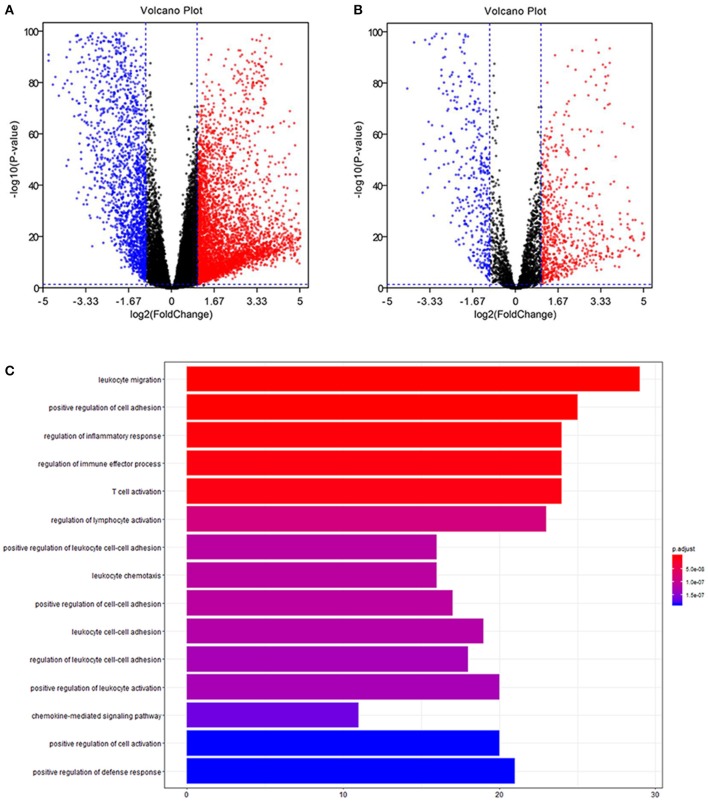
Differentially expression and functional enrichment. **(A)** Volcano chart of differentially expressed genes. **(B)** Volcano chart of immune differentially expressed genes. **(C)** Barplot chart for functional enrichment analysis.

Volcano plots of 21,205 mRNAs and 3,627 immune genes are presented in Figures 1A,B. There were 265 up-regulated and 185 down-regulated immune genes in differentially expressed analyses.

### Functional Enrichment Analyses

**Figure 2:**
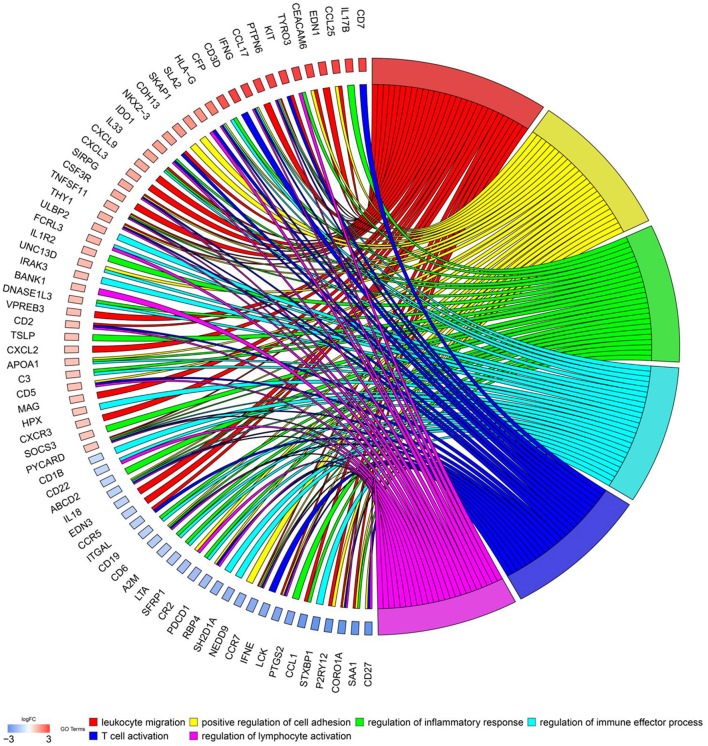
Chord chart of prognostic genes.

To explore biological functions of immune genes, Gene Ontology (GO) and Kyoto Encyclopedia of Genes and Genomes (KEGG) were performed. Bar plot (Figure 1C), GO chord chart (Figure 2), and KEGG chord plot (Supplementary Figure 2) showed that biological functions of immune genes were mainly enriched in leukocyte migration, positive regulation of cell adhesion, regulation of inflammatory response, regulation of immune effector process, T cell activation, regulation of lymphocyte activation, positive regulation of leukocyte cell–cell adhesion, leukocyte chemotaxis, positive regulation of cell–cell adhesion, and leukocyte cell–cell adhesion. The top five KEGG items were as follows: cytokine–cytokine receptor interaction, hematopoietic cell lineage, viral protein interaction with cytokine and cytokine receptor, human T cell leukemia virus 1 infection, and PI3K–Akt signaling pathway.

### Immune Regulatory Network

**Figure 3:**
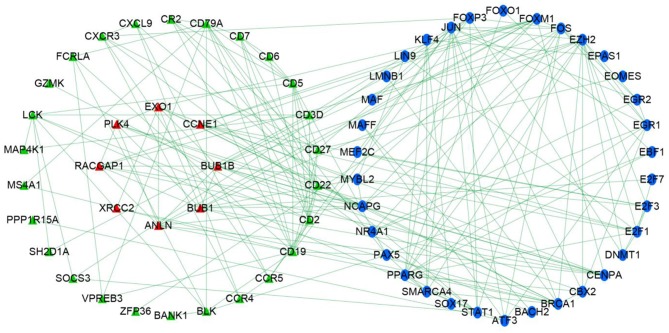
Immune gene regulatory network chart.

Univariate Cox regression determined 179 prognostic genes for DFS. The current research adopted three methods to explore the relationship between immune genes and transcription factors. First, with thresholds of correlation coefficient >0.5 and *P* < 0.01, the current study identified transcription factors that were highly correlated with prognostic immune genes. Second, prognostic immune genes and their highly correlated transcription factors were put in STRING database (medium confidence, 0.70) to explore relationships among prognostic immune genes and transcription factors. Finally, Cytoscape v3.6.1 was used to develop an immune regulatory network (Figure 3) on 34 immune genes and 34 transcription factors (24).

### Construction of Prognostic Model

**Figure 4:**
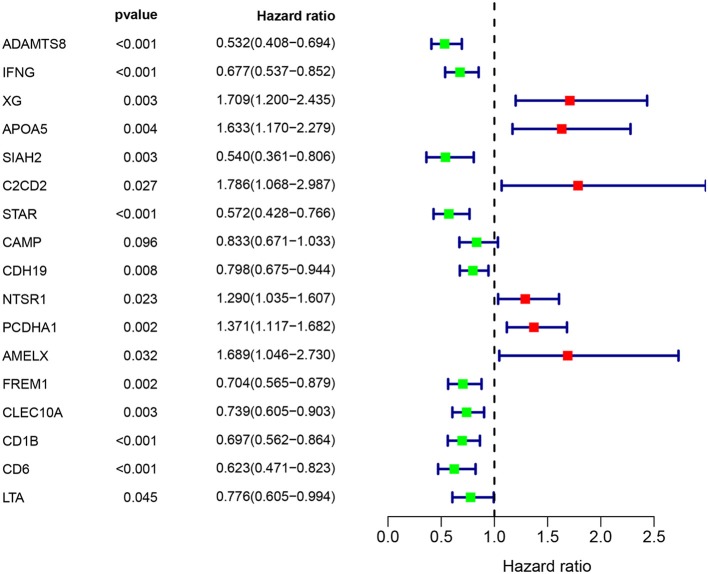
Immune gene survival forest chart.

Multivariate Cox regression identified 17 genes as independent influence factors for BC (Table 2 and Figure 4). The formula of prognostic model was as follows:

$$\begin{array}{l} \text{prognostic score}=\left(-0.499\;*\text{ ADAMTS}8\right)+\left(-0.698\;*\text{ IFNG}\right)\\ +\;\left(0.790\;*\text{ XG}\right)+\left(0.645\;*\text{ APOA}5\right)\\ +\;\left(-0.901\;*\text{ SIAH}2\right)+\;\left(1.117\;*\text{ C}2\text{CD}2\right)\\ +\;\left(-0.507\;*\text{ STAR}\right)+\left(-0.321\;*\text{ CAMP}\right)\\ +\;\left(-0.261\;*\text{ CDH}19\right)+\left(0.382\;*\text{ NTSR}1\right)\\ +\;\left(0.331\;*\text{ PCDHA}1\right)+\left(0.706\;*\text{ AMELX}\right)\\ +\;\left(-0.655\;*\text{ FREM}1\right)+\left(1.082\;*\text{ CLEC}10\text{A}\right)\\ +\;\left(-0.497\;*\text{ CD}1\text{B}\right)+\left(-0.909\;*\text{ CD}6\right)\\ +\;\left(0.620\;*\text{ LTA}\right). \end{array}$$

**Figure 5:**
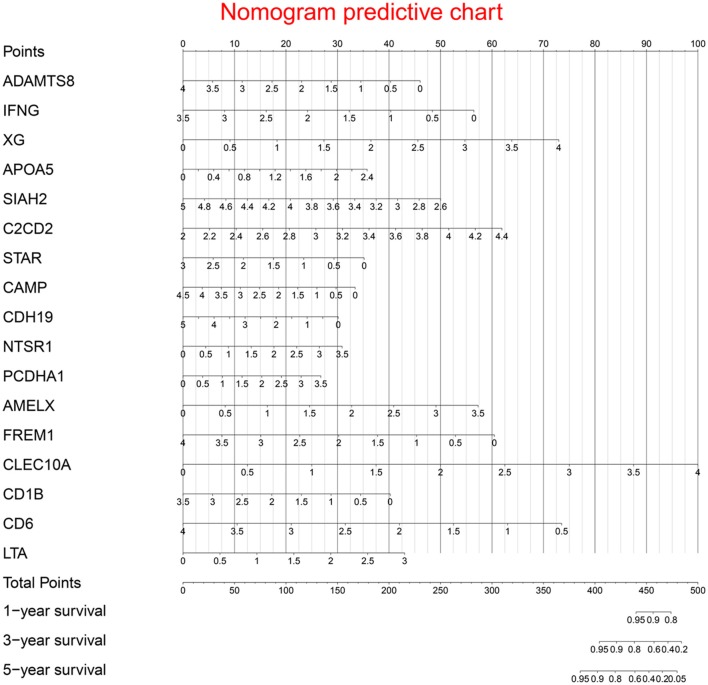
Prognostic nomogram chart.

Prognostic nomogram is shown in Figure 5.

**Table 2:** Information of prognostic immune genes.

	**Univariate analysis**			**Multivariate analysis**			
**Immune gene**	**HR**	**95% CI**	***P***	**Coefficient**	**HR**	**95% CI**	***P***
ADAMTS8 (high/low)	0.601	0.453	0.796	−0.499	0.607	0.444–0.832	0.002
IFNG (high/low)	0.627	0.472	0.833	−0.698	0.497	0.335–0.739	<0.001
XG (high/low)	1.373	1.036	1.819	0.790	2.203	1.520–3.193	<0.001
APOA5 (high/low)	1.465	1.084	1.979	0.645	1.906	1.331–2.730	<0.001
SIAH2 (high/low)	0.686	0.519	0.908	−0.901	0.406	0.264–0.624	<0.001
C2CD2 (high/low)	1.327	1.004	1.753	1.117	3.056	1.742–5.361	<0.001
STAR (high/low)	0.631	0.477	0.835	−0.507	0.602	0.432–0.839	0.003
CAMP (high/low)	0.740	0.559	0.979	−0.321	0.725	0.580–0.907	0.005
CDH19 (high/low)	0.676	0.510	0.895	−0.261	0.771	0.648–0.916	0.003
NTSR1 (high/low)	1.410	1.068	1.860	0.382	1.465	1.162–1.847	<0.001
PCDHA1 (high/low)	1.475	1.116	1.950	0.331	1.392	1.125–1.723	0.002
AMELX (high/low)	1.488	1.019	2.172	0.706	2.026	1.236–3.322	0.005
FREM1 (high/low)	0.678	0.512	0.898	−0.655	0.520	0.362–0.747	<0.001
CLEC10A (high/low)	0.699	0.528	0.925	1.082	2.950	1.756–4.957	<0.001
CD1B (high/low)	0.729	0.550	0.966	−0.497	0.608	0.407–0.909	0.015
CD6 (high/low)	0.619	0.467	0.821	−0.909	0.403	0.215–0.755	0.005
LTA (high/low)	0.730	0.551	0.967	0.620	1.860	1.048–3.300	0.034

*The medians of gene expression values were used as cutoff values to stratify gene expression values into high expression group (as value 1) and low expression group (as value 0). HR, hazard ratio; CI, confidence interval*.

Survival curves of prognostic genes are shown in Supplementary Figure 3. Supplementary Figures 4, 5 are predictive value distribution chart and survival status scatterplot, respectively.

### Clinical Performance of Model Cohort

**Figure 6:**
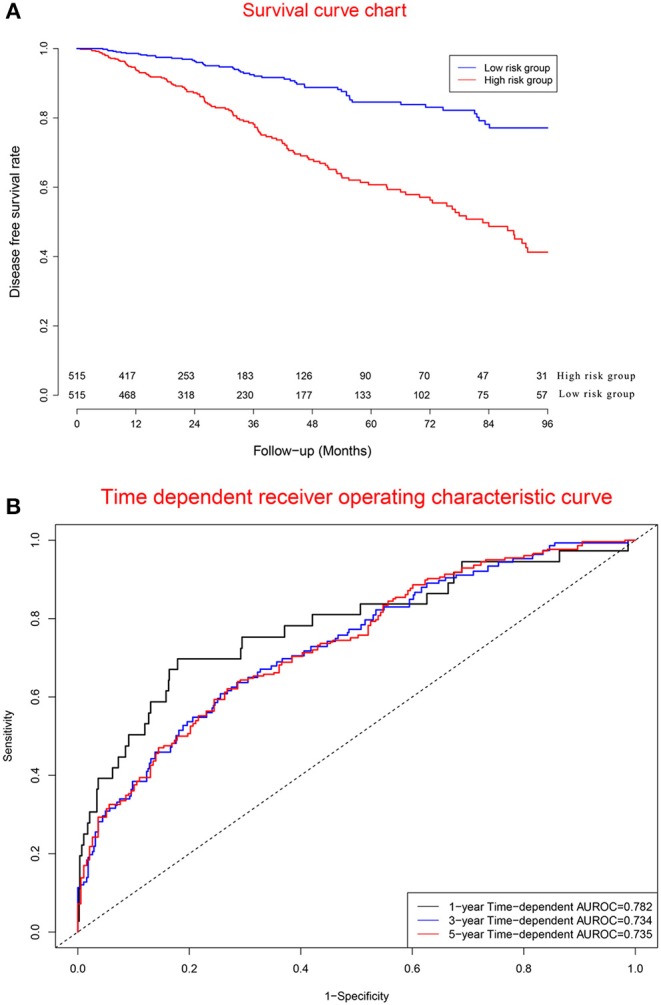
Clinical performance in model cohort. **(A)** Survival curves for high risk group and low risk group. **(B)** Time-dependent receiver operating characteristic curves.

Figure 6A displays survival curves in the high-risk group and low-risk group divided by the median of prognostic scores. Figure 6B demonstrates that concordance indexes were 0.782, 0.734, and 0.735 for 1-, 3-, and 5-year survival, respectively. Supplementary Figure 6 shows calibration curves of the model cohort.

### Clinical Performance of Validation Cohort

**Figure 7:**
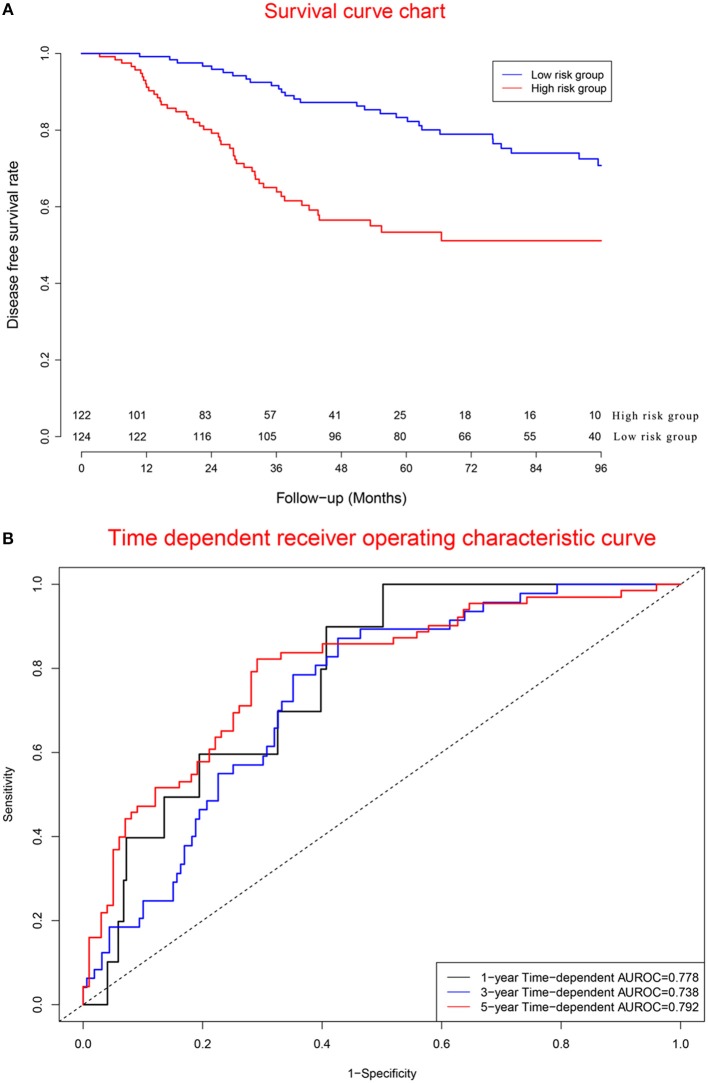
Clinical performance in validation cohort. **(A)** Survival curves for high risk group and low risk group. **(B)** Time-dependent receiver operating characteristic curves.

Figure 7A displays survival curves in the high-risk group and low-risk group. Figure 7B demonstrates that concordance indexes were 0.778, 0.738, and 0.792 for 1-, 3-, and 5-year survival, respectively. Supplementary Figure 7 shows calibration curves of the validation cohort.

### Artificial Intelligence Survival Prediction System

**Figure 8:**
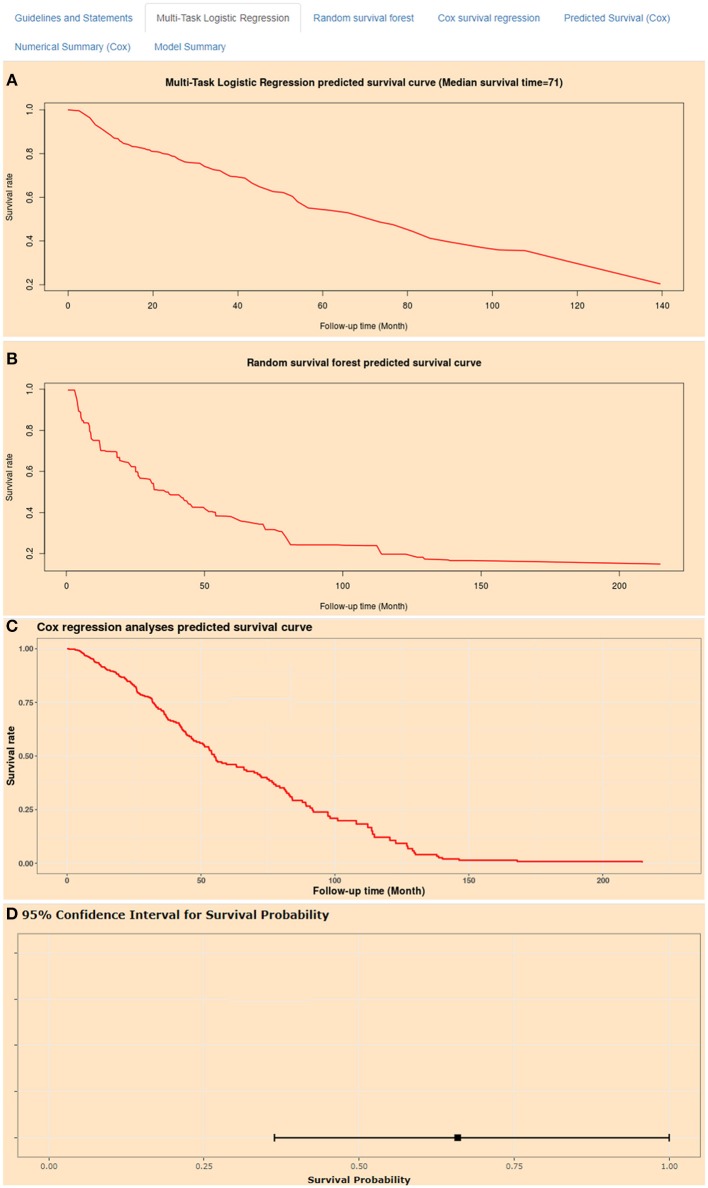
Home page of Smart Cancer Survival Predictive Predictive System. **(A)** Multi-Task logistic regression predicted survival curves. **(B)** Random survival forest predicted survival curves. **(C)** Cox survival regression predicted survival curves. **(D)** Cox survival regression predicted mortality percentage and 95% confidence interval.

Artificial intelligence survival prediction system was constructed to provide individual mortality risk prediction for BC patients (Figure 8). This tool could provide three individual mortality risk predictive curves by using random survival forest algorithm (Figure 8A), multitask logistic regression algorithm (Figure 8B), and Cox survival regression algorithm (Figure 8C). Artificial intelligence survival prediction system is available at https://zhangzhiqiao7.shinyapps.io/Smart_Cancer_Survival_Predictive_System_16_BC_C1005/.

### Gene Survival Analysis Screen System

The Gene Survival Analysis Screen System was constructed for exploratory research of immune genes (Supplementary Figure 8). The Gene Survival Analysis Screen System is available at https://zhangzhiqiao8.shinyapps.io/Gene_Survival_Subgroup_Analysis_16_BC_C1005/.

### Independence Assessment

Prognostic signature, AJCC PT, and AJCC PN were independent risk factors for DFS in the model dataset (Table 3). In the validation dataset, prognostic signature was proven to be an independent risk factor for DFS.

**Table 3:** Results of Cox regression analyses.

	**Univariate analysis**			**Multivariate analysis**			
	**HR**	**95% CI**	***P***	**Coefficient**	**HR**	**95% CI**	***P***
TCGA cohort (*n* = 1030)							
Age (high/low)	1.573	1.189–2.080	0.002	0.363	1.438	1.080–1.915	0.013
AJCC PT (T3-4/T1-2)	1.936	1.419–2.643	<0.001	0.562	1.754	1.279–2.406	<0.001
AJCC PN (N2-3/N0-1)	2.138	1.590–2.875	<0.001	0.704	2.021	1.490–2.741	<0.001
Prognostic model (high/low)	3.285	2.423–4.453	<0.001	1.146	3.147	2.308–4.291	<0.001
GSE31448 cohort (*n* = 246)							
Age (high/low)	1.076	0.691–1.675	0.747	0.159	1.173	0.740–1.858	0.498
AJCC PT (T3-4/T1-2)	1.471	0.904–2.396	0.121	0.273	1.313	0.790–2.184	0.293
AJCC PN (N2-3/N0-1)	1.448	0.923–2.271	0.107	0.337	1.401	0.883–2.221	0.152
Prognostic model (high/low)	2.850	1.791–4.535	<0.001	1.057	2.878	1.806–4.586	<0.001

*The median of prognostic model scores was used as the cutoff value to stratify gastric cancer patients into high-risk group and low risk group. AJCC, the American Joint Committee on Cancer; HR, hazard ratio; CI, confidence interval*.

### Clinical Correlation Analyses

**Figure 9:**
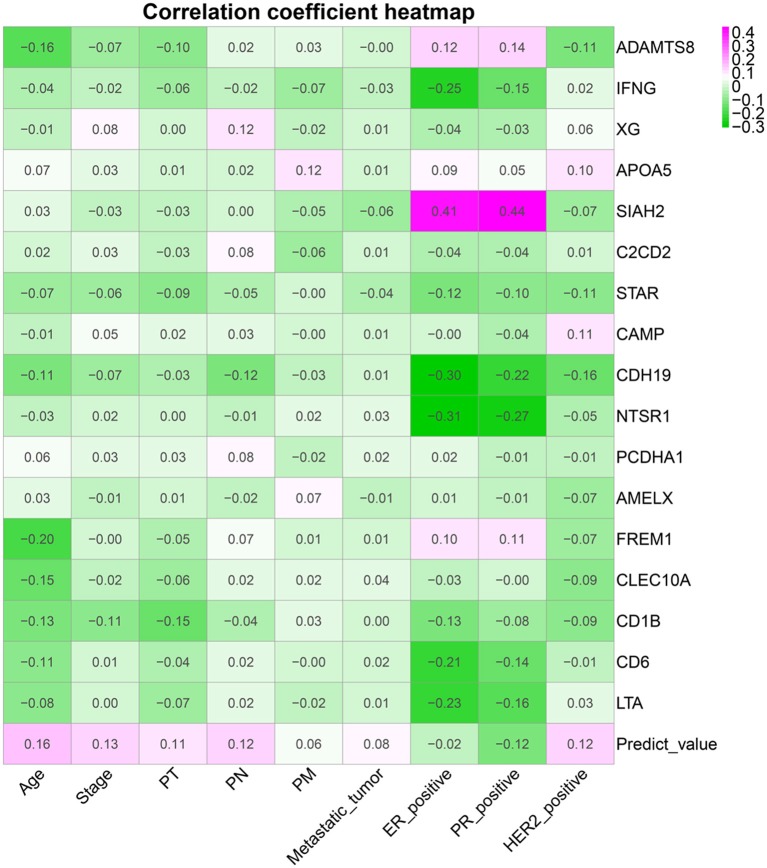
Clinical variable correlation coefficient heatmap.

Figure 9 shows a correlation coefficient heatmap between prognostic genes and clinical variables. Supplementary Figure 9 presents correlation significance heatmap between prognostic genes and clinical variables.

### Tumor Immune Infiltration Correlation Analyses

**Figure 10:**
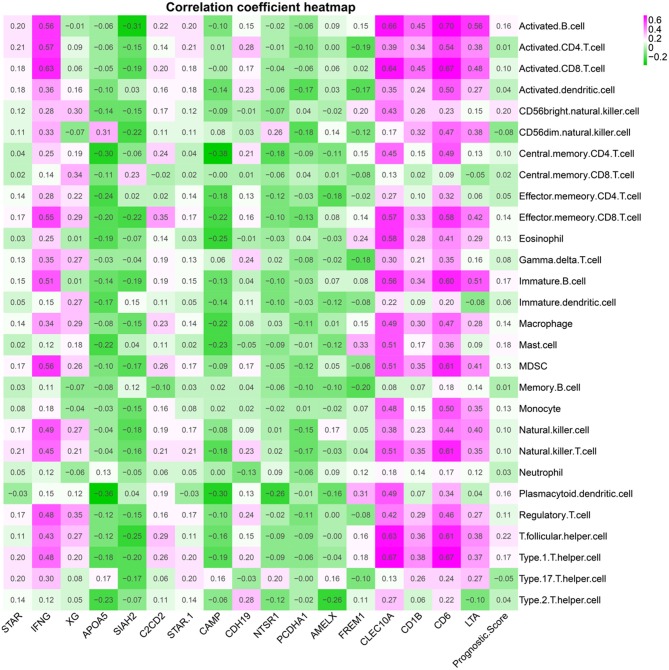
Immune gene correlation coefficient heatmap.

Figure 10 presents correlation coefficient heatmap between tumor immune infiltration and prognostic genes. Supplementary Figure 10 presents correlation significance heatmap between tumor immune infiltration and prognostic genes.

### Tumor Immune Infiltration

**Figure 11:**
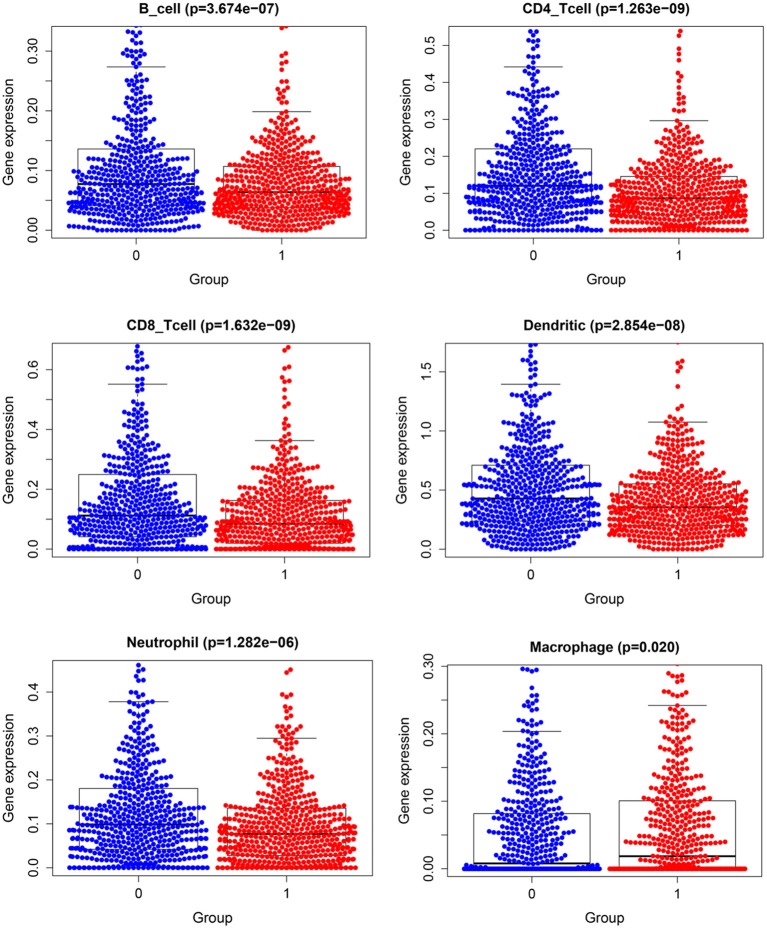
Expression of tumor immune–infiltrating cells.

**Figure 12:**
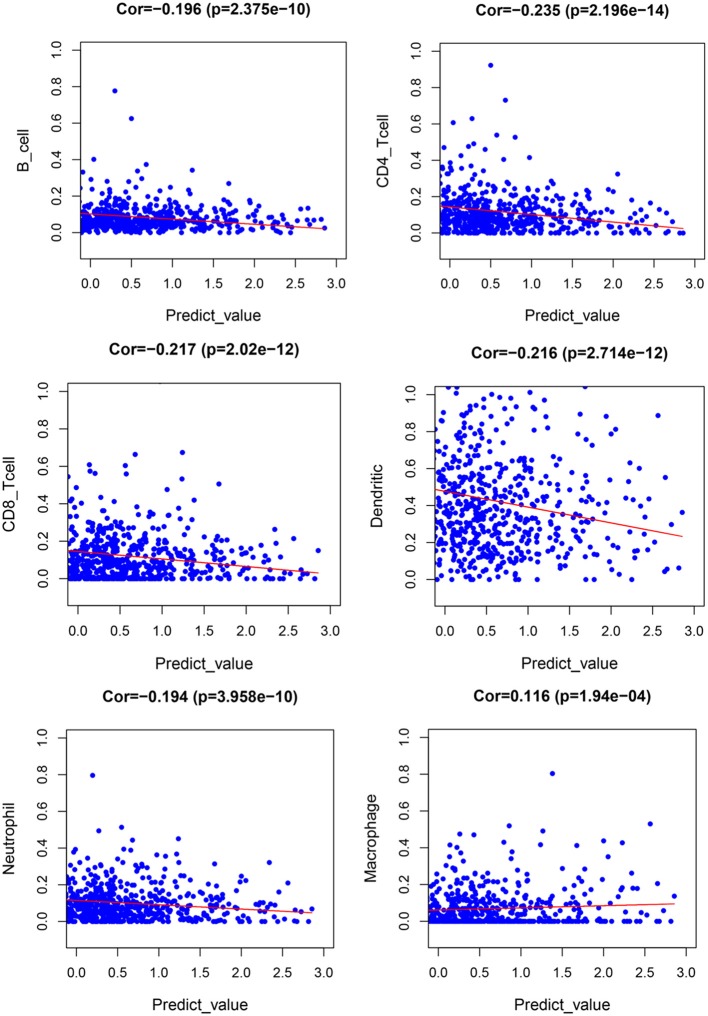
Scatterplot of tumor-infiltrating immune cells and prognostic signature.

Figure 11 demonstrates expression levels of six tumor immune infiltration in the high-risk group and low-risk group. Figure 12 presents scatterplots between six tumor immune infiltrations and prognostic score.

### Gene Differential Expression Between Normal Samples and Tumor Samples

To demonstrate the gene differential expression between normal samples and tumor samples at the molecular level, the current study performed group differential expression analyses between normal samples and tumor samples obtained from TCGA database. There were 1,109 tumor samples and 113 normal samples for group differential expression analyses. Supplementary Figure 11 presents the gene differential expression between normal samples and tumor samples at the molecular level.

### Clinical Performance in Different Cancers

To explore the clinical performance of the current prognostic model for other cancers, four tumor datasets were obtained from TCGA database as external validation datasets. There were 348 patients in the hepatocellular carcinoma dataset, 265 patients in the colorectal cancer dataset, 494 patients in the lung cancer dataset, and 370 patients in the ovarian cancer dataset. The prognostic scores in external validation datasets were calculated according to the previous formula derived from the model dataset. Survival curve analyses indicated good diagnostic performance of the current prognostic model for hepatocellular carcinoma, colorectal cancer, lung cancer, and ovarian cancer (Supplementary Figure 12), suggesting that the current prognostic model might be useful for other malignant tumors.

### External Validation of Accuracy and Clinical Validity

To validate the accuracy and clinical validity of the current prognostic model in other external validation dataset, hepatocellular carcinoma dataset, colorectal cancer dataset, lung cancer dataset, and ovarian cancer dataset were downloaded from TCGA database. These four malignant-tumor datasets were merged into one joint dataset as external validation dataset with 1,640 tumor patients. Supplementary Figure 13A demonstrates that concordance indexes were 0.832, 0.781, and 0.778 for 1-, 3-, and 5-year survival, respectively. Supplementary Figure 13B suggests that the current prognostic model could distinguish tumor patients with high mortality risk from those with low mortality risk. Calibration curves of external validation dataset showed good accordance between actual mortality percentage and predicted mortality percentage (Supplementary Figure 14).

## Discussion

The current research determined 17 immune genes as prognostic biomarkers for DFS. Then the current research depicted regulatory relationships among transcription factors and immune genes through correlation analyses and STRING database. Based on these 17 immune genes, the current research created a prognostic nomogram to predict the DFS for BC patients. Based on the previous prognostic nomogram, the current research developed two artificial intelligence survival predictive systems for individual mortality risk prediction. These two artificial intelligence survival predictive systems were helpful to provide precise individual mortality risk prediction and improve individual treatment decision-making.

Several prognostic models have been built for predicting the prognosis in BC patients (11–13). The previous prognosis models could only provide the mortality curves for two groups of tumor patients with different characteristics, failing to provide the individual mortality curve for a special patient. The progress of artificial intelligence algorithms provides necessary basic conditions for the realization of individualized mortality risk prediction of cancer patients. Random survival forest algorithm (25–27), multitask logistic regression (28, 29), and Cox survival regression algorithm (30) have been proposed and used to improve the predictive performance of prognostic models. Based on three artificial intelligence algorithms above, we develop an artificial intelligence survival predictive system. Our artificial intelligence survival predictive system could display three individual mortality risk predictive curves by using random survival forest algorithm, multitask logistic regression algorithm, and Cox survival regression algorithm. At present, there are few prognostic models that can provide individual mortality risk prediction. The current study provides an interesting and feasible way for the transformation and application of artificial intelligence algorithm in the field of medicine.

Tumor immune infiltration acted an important role in oncogenesis and prognosis (7, 31). Immune genes could be used to predict the prognosis of BC patients (32, 33). Three prognostic models have been developed to predict the prognosis for BC patients (11–13). Compared with three previous prognostic models, the current prognostic model could provide individualized mortality risk prediction and online calculation function, which were of great significance for clinical application by patients and clinicians.

Biological processes of immune genes were explored via TISIDB databases (http://cis.hku.hk/TISIDB/index.php). Top biological processes of CD1b molecule (CD1B) were adaptive immune response, antigen processing and presentation, and antigen processing and presentation via major histocompatibility complex class Ib. Top biological processes of lymphotoxin α (LTA) were adaptive immune response, lymphocyte-mediated immunity, leukocyte-mediated immunity, and inflammatory response to antigenic stimulus. Top biological processes of CD6 molecule (CD6) were immunological synapse formation, cell recognition, acute inflammatory response, and inflammatory response to antigenic stimulus. Top biological processes of cathelicidin antimicrobial peptide (CAMP) were cell killing, antibacterial humoral response, innate immune response in mucosa, mucosal immune response, and organ- or tissue-specific immune response. Top biological processes of interferon γ9 (IFNG) were cell killing, neutrophil homeostasis, leukocyte homeostasis, and neutrophil apoptotic process. Top biological processes of ADAM metallopeptidase with thrombospondin type 1 motif 8 (ADAMTS8) were phosphate ion transport, anion transport, and inorganic anion transport. Top biological processes of apolipoprotein A-V (APOA5) were receptor-mediated endocytosis, tissue regeneration, and positive regulation of receptor-mediated endocytosis. Top biological processes of siah E3 ubiquitin protein ligase 2 (SIAH2) were proteasomal protein catabolic process, regulation of cysteine-type endopeptidase activity involved in apoptotic process, and negative regulation of cysteine-type endopeptidase activity involved in apoptotic process. Top biological processes of steroidogenic acute regulatory protein (STAR) were response to molecule of bacterial origin, response to oxidative stress, and response to reactive oxygen species. Top biological processes of cadherin 19, type 2 (CDH19), were homophilic cell adhesion via plasma membrane adhesion molecules and cell–cell adhesion via plasma-membrane adhesion molecules. Biological processes of C-type lectin domain family 10, member A (CLEC10A), were adaptive immune response.

ADAMTS8 was related with poor prognosis for breast invasive ductal carcinoma patients (34). ADAMTS8 could regulate invasion and apoptosis of hepatocellular carcinoma through ERK signaling pathway (35). The interaction between CD4^+^ T cells and lung cancer cells could up-regulate expression of DNMT and methylation of IFNG promoter (36). CpG methylation of IFNG gene could induce immunosuppression of tumor-infiltrating lymphocytes (37). High expression level of XG in Ewing sarcoma cell line could promote tumor migration and invasiveness (38). Highly expressed SIAH2 was associated with poor progression-free survival after tamoxifen treatment (39). SIAH2 participated in the regulation of EAF2 polyubiquitin in prostate cancer cells as E3 ligase of EAF2 polyubiquitination (40). Cathelicidin antimicrobial peptide directly activated exchange protein, which regulated migration and apoptosis of BC cells (41). Interleukin 24 enhanced apoptosis of BC cell via cAMP-dependent PKA pathway (42). Low expression of NTSR1 was associated with non-invasive growth of colorectal cancer (43). Interaction of CLEC10A with macrophages and dendritic cells might play an important role in tumor progression (44). As a functional ligand of CLEC10A, sv6D could induce the maturation of immune cells (45). Variation of STXBP6 might affect the response of TNF-α inhibitors in rheumatoid arthritis patients (46). There was a significant correlation between LTA RS909253GA genotype and the development of Asian gastric cancer (47). These previous studies revealed possible immune regulatory mechanisms and biological roles of previous 17 immune genes in tumorigenesis and progression.

Toll-like receptor–activated plasma-like dendritic cells inhibited growth of BC cells (48). CD56 enhanced formation of cytotoxic immune synapses and strengthened sensitivity of cytotoxicity mediated by natural killer cells (49). CD4 and CD8 T cell tumor infiltration driven by HER2-dendritic cells improved survival of BC mice (50). CD4^+^ T cells inhibited CD8^+^ T cell failure at the initiation stage of immune response in BC (51). V delta 2^+^ gamma delta T lymphocyte had cytotoxicity to MCF 7 BC cells (52). High expression of SEMA4C was correlated with the proliferation of tumor cells and the aggregation of macrophages in BC (53). Interleukin 32θ suppressed the growth of BC by regulating CCL18 secreted by macrophages (54). Macrophage adhesion regulated by integrin induces lymphovascular dissemination in BC (55). CCL5 induced recurrence of BC via aggregation of macrophages in residual tumors (56). High expression of mast cells induced the tumor size and the incidence of spontaneous metastasis in BC mice (57). Tumor-infiltrating myeloid-derived suppressor cells (MDSCs) was related with therapeutic effect and prognosis of neoadjuvant chemotherapy of BC (58). Neutrophil–lymphocyte ratio could predict prognosis of triple-negative BC patients (59). Interleukin 10 and interleukin 2 promoted proliferation and cytotoxicity of CD8+T cells (60).

Advantages of current research: First, two artificial intelligence survival predictive systems were developed based on immune genes for BC patients. These two tools could provide online individual mortality risk prediction and provide valuable prognostic information for optimizing individual treatment decision. Second, artificial intelligence survival prediction system provided three individual mortality risk predictive curves based on different artificial intelligence algorithms, providing different valuable individual mortality curves as the references of individual medical decision-making. Third, artificial intelligence survival prediction system provided predicted median survival time and 95% confidence interval of predicted mortality, which were of clinical practical values for optimizing individual medical decision-making.

Shortcomings of current research: First, the current research explored potential biological functions and regulatory mechanisms of immune genes in BC based on public databases, but the conclusion was not validated by confirmative experimental studies. Second, estrogen receptor, progesterone receptor, and erb-b2 receptor tyrosine kinase 2 are closely related to prognosis of BC patients. Subgroup studies based on these biomarkers are helpful to provide more accurate individual mortality risk prediction for BC patients in different subgroups. Third, the current studies did not include and analyze the impacts of several clinical factors, such as radiotherapy, chemotherapy, and targeted drug therapy, which should be taken into account for future studies. Fourth, the calculation process of artificial intelligence algorithms are too complex to perform and cannot be present through conventional formula. The operation process of artificial intelligence algorithm is opaque, just like a black box, which limits the clinical application and verification of artificial intelligence algorithm. Because it is difficult for artificial intelligence algorithm to perform repeated verification research, we provided three different artificial intelligence algorithms as references for each other. As the inherent deficiency of artificial intelligence, opaque computing process and lack of verification research need to be solved by future artificial intelligence algorithm research. Fifth, the current study identified 17 immune genes were correlated with prognosis of BC patients. However, the associations of these immune genes with tumor heterogeneity and tumor resistance were still unclear. Further basic immune research is helpful to clarify the associations of these immune genes with tumor heterogeneity and tumor resistance. Sixth, although the current study demonstrated the gene differential expression between normal samples and tumor samples, the current study was lack of external validation at the cell level and the animal model level. Further validation studies at the cell level and the animal model level were helpful to ascertain the differences of immune regulatory mechanism of BC patients compared with normal people.

In conclusion, comprehensive bioinformatics identified 17 immune genes as potential prognostic biomarkers, which might be potential candidates of immunotherapy targets in BC patients. The current study depicted regulatory network between transcription factors and immune genes, which was helpful to deepen the understanding of immune regulatory mechanisms for BC cancer. Two artificial intelligence survival predictive systems are available at https://zhangzhiqiao7.shinyapps.io/Smart_Cancer_Survival_Predictive_System_16_BC_C1005/ and https://zhangzhiqiao8.shinyapps.io/Gene_Survival_Subgroup_Analysis_16_BC_C1005/. These novel artificial intelligence survival predictive systems will be helpful to improve individualized treatment decision-making.

## Data Availability Statement

Publicly available datasets were analyzed in this study. This data can be found here: https://zhangzhiqiao8.shinyapps.io/Gene_Survival_Subgroup_Analysis_16_BC_C1005/.

## Ethics Statement

The studies in TCGA database and GEO database have received ethical approval from ethics committees of their respective research institutes. These studies obtained informed consent from patients before admission. The current study is a second study based on public datasets from TCGA database and GEO database. Details of all patients in public datasets have been anonymously processed and therefore the current research does not involve patients' privacy information. This study was performed according to public database policy and declaration of Helsinki. Ethical approval and informed consent were not applicable.

## Author Contributions

ZZ, JD, JL, and TH: conceptualization, methodology and resources. ZZ, JD, JL, and TH: investigation, data curation, formal analysis, validation, software, project administration, and supervision. ZZ, and JD: writing and visualization. ZZ: funding acquisition.

### Conflict of Interest

The authors declare that the research was conducted in the absence of any commercial or financial relationships that could be construed as a potential conflict of interest.

## Abbreviations

BC: breast cancer
TCGA: The Cancer Genome Atlas
GEO: the Gene Expression Omnibus
ROC: receiver operating characteristic
DFS: disease free survival
HR: hazard ratio
CI: confidence interval
AJCC: the American Joint Committee on Cancer
SD: standard deviation.
