# Supplementary material for: Bioinformatics Identified 17 Immune Genes as Prognostic Biomarkers for Breast Cancer: Application Study Based on Artificial Intelligence Algorithms
Source: Front Oncol. 2020 Mar 31;10:330. doi: 10.3389/fonc.2020.00330 (PMC7137378; doi:10.3389/fonc.2020.00330)
Supplement: Supplementary file 1 [file Data_Sheet_1.pdf]

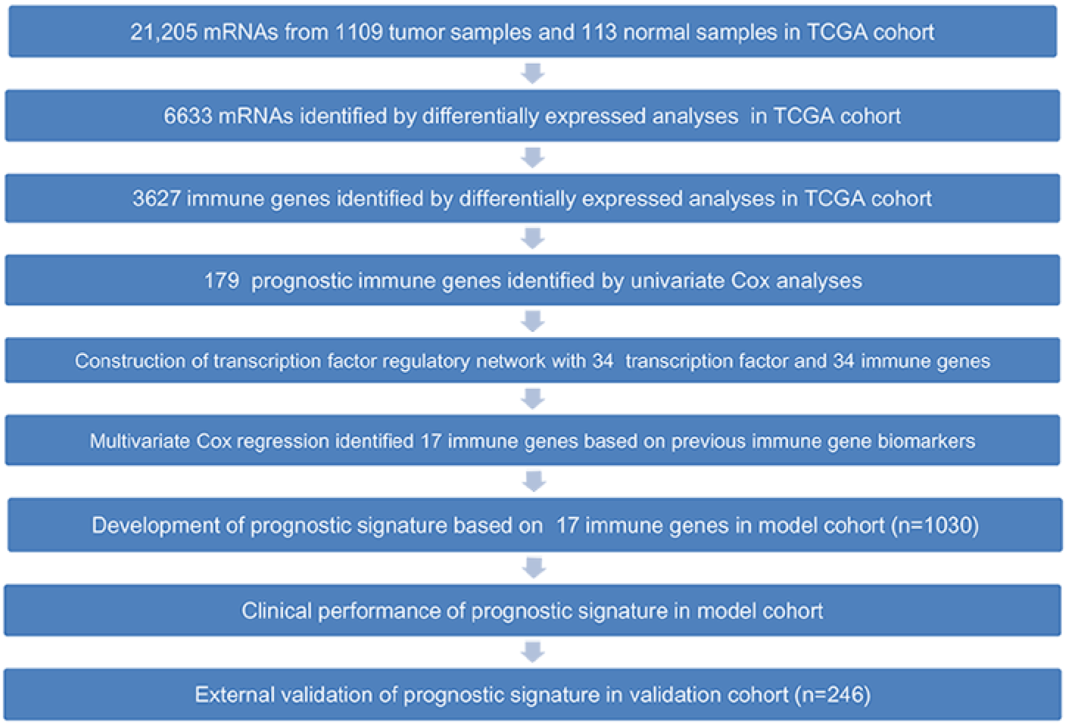

Supplementary Figure1. Flow chart in current study

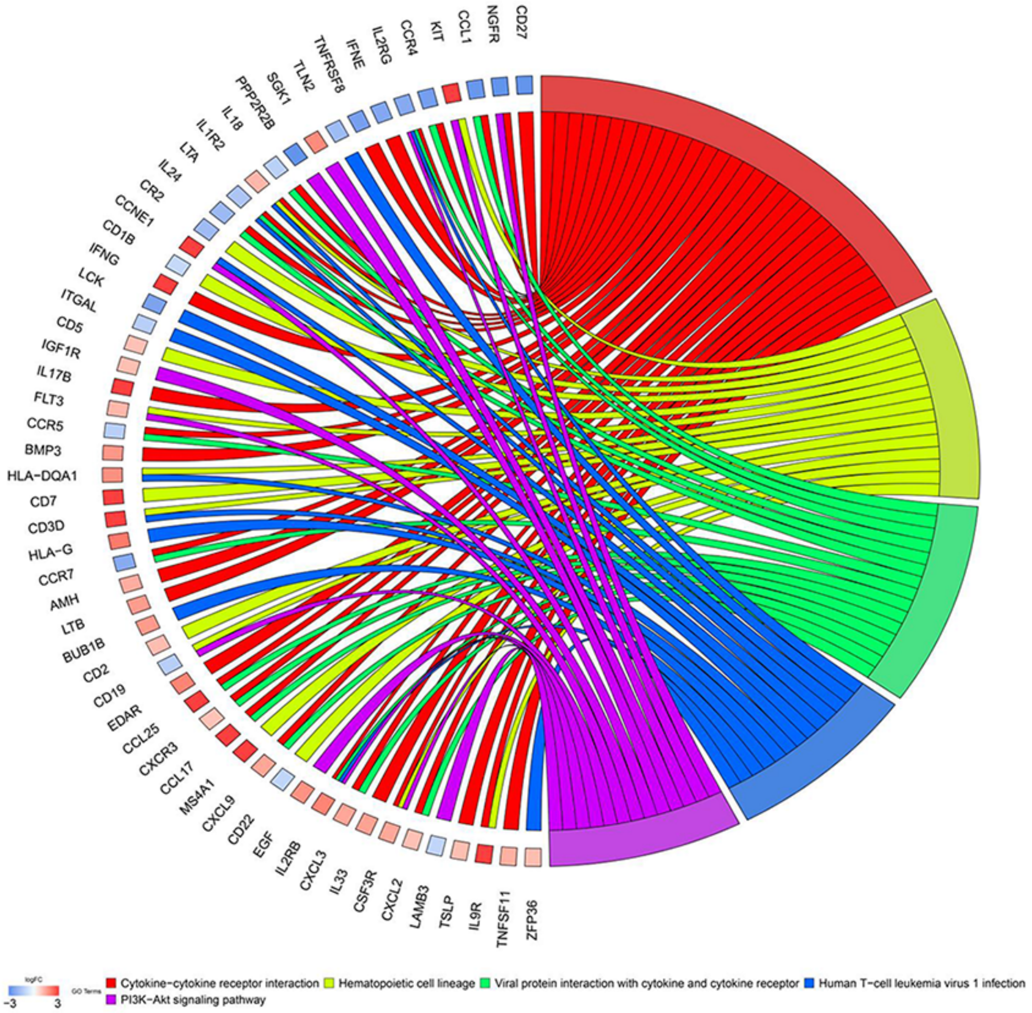

Supplementary Figure 2. KEGG function enrichment chord chart

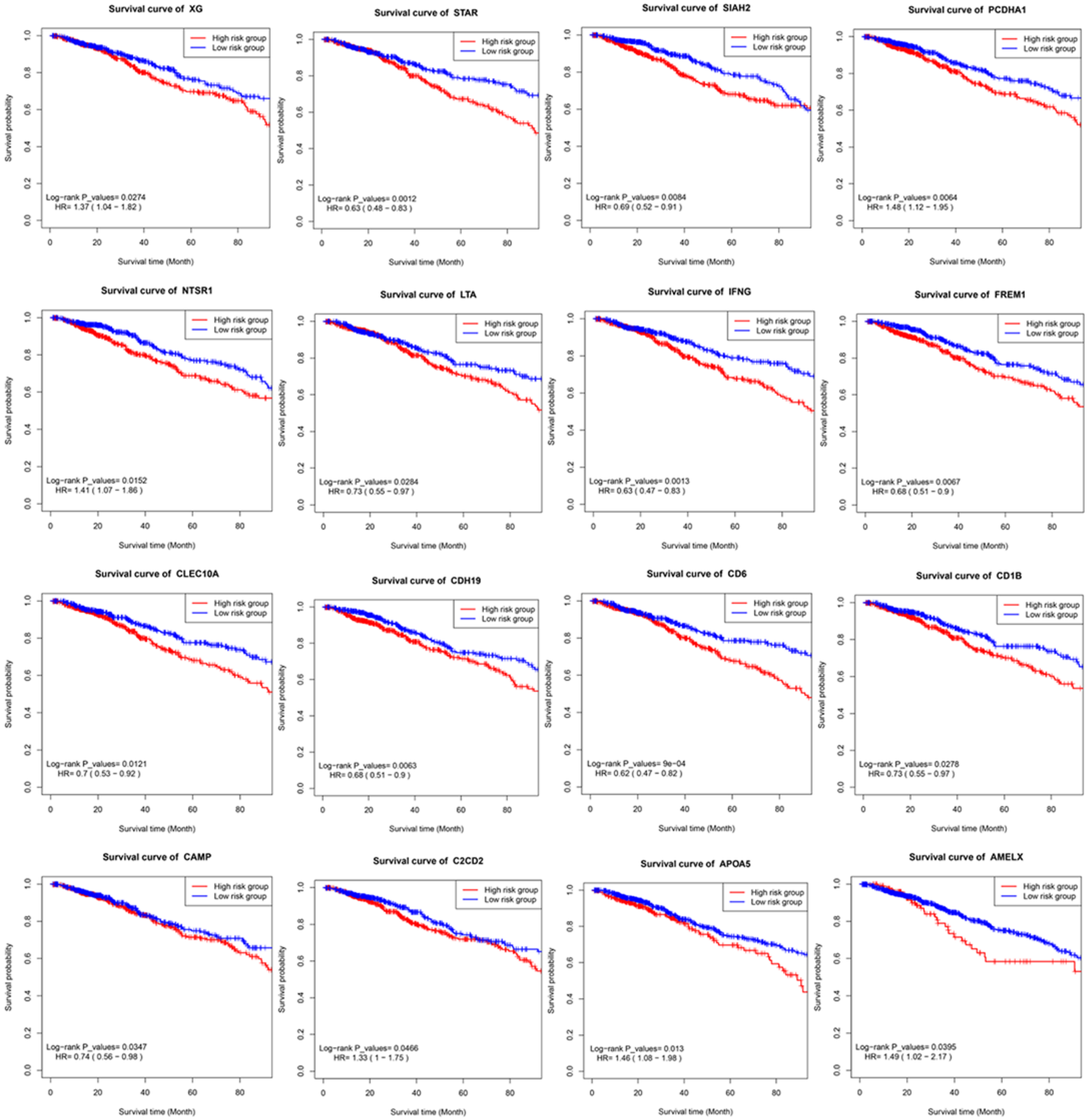

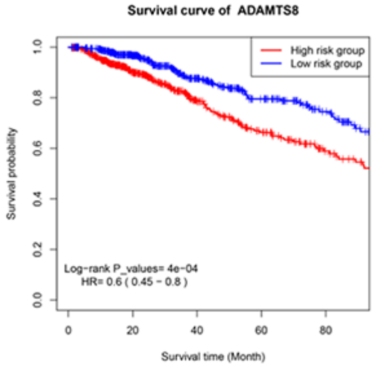

Supplementary Figure 3. Survival curves of immune genes

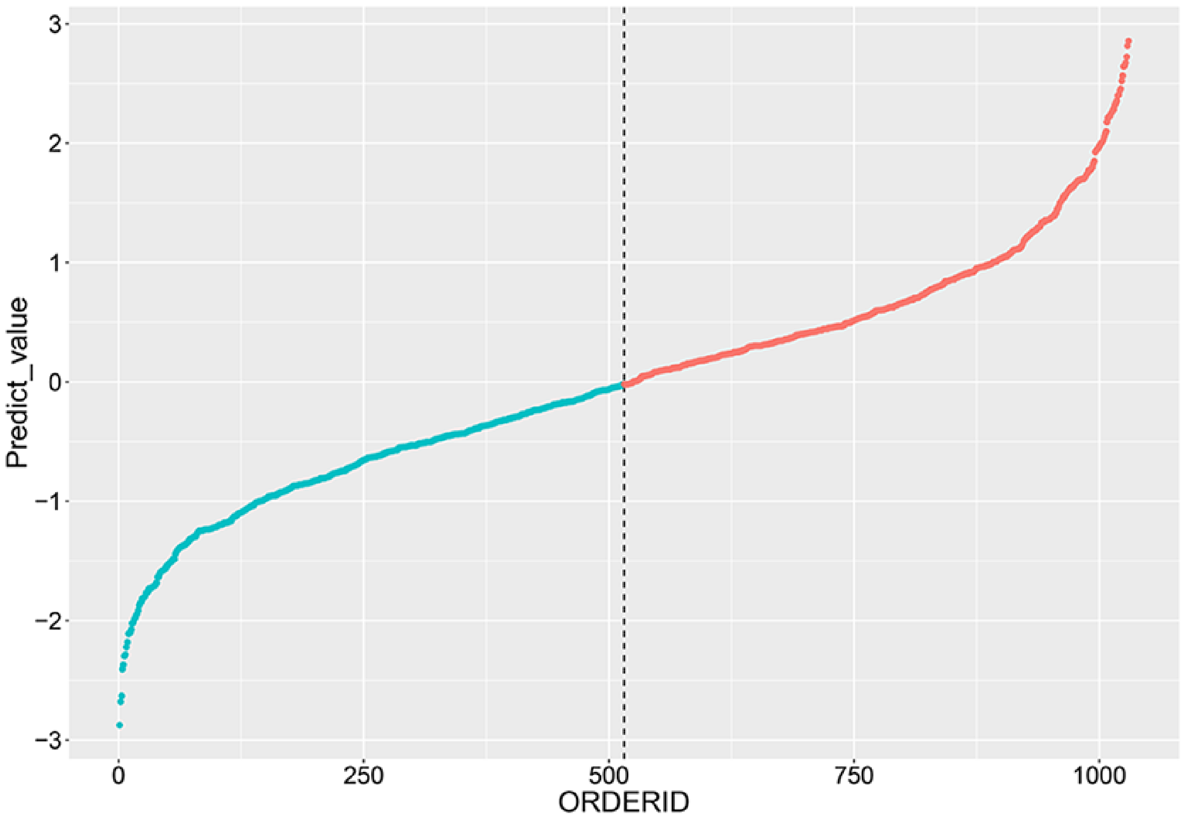

Supplementary Figure 4. Predictive value distribution chart

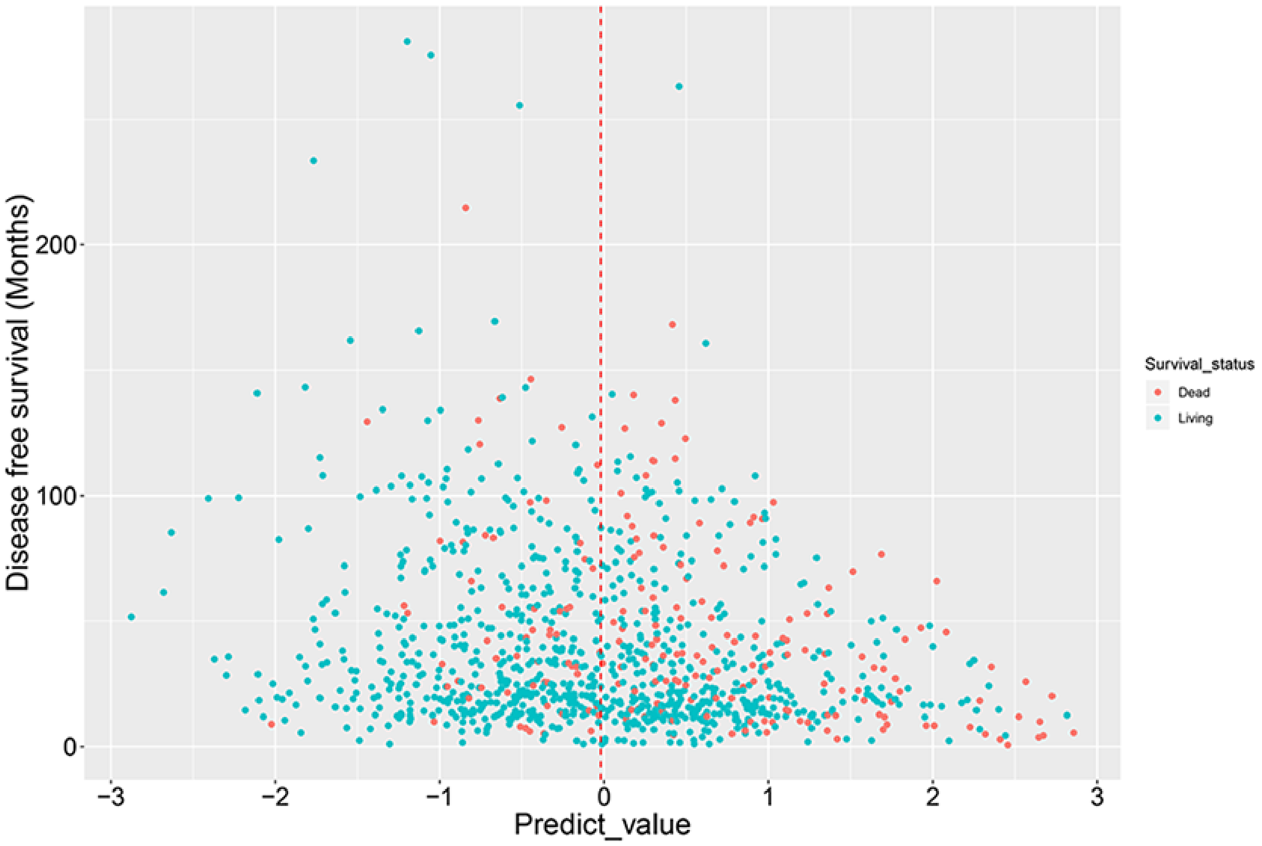

Supplementary Figure 5. Survival status scatter plot

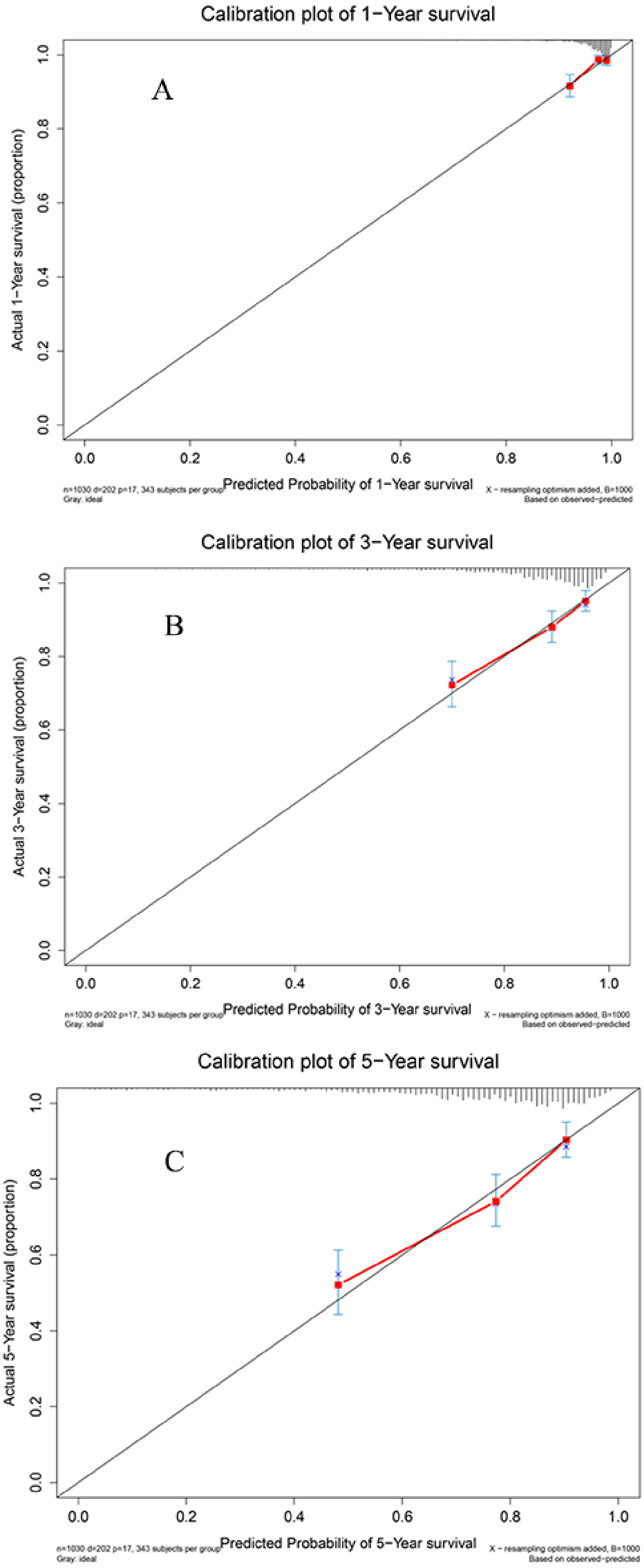

Supplementary Figure 6. Calibration curves for model cohort: (A) Calibration curve for 1-year survival; (B) Calibration curve for 3-year survival; (C) Calibration curve for 5-year survival

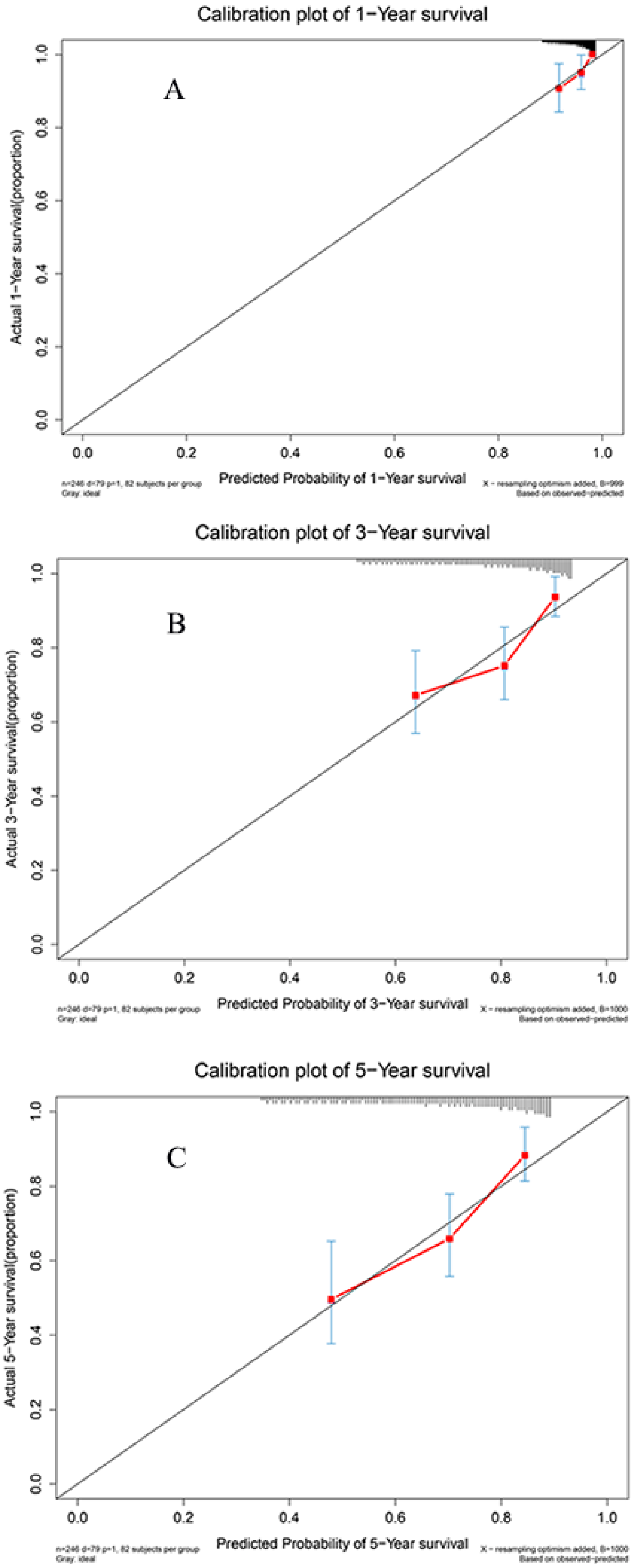

Supplementary Figure 7. Calibration curves for validation cohort: (A) Calibration curve for 1-year survival; (B) Calibration curve for 3-year survival; (C) Calibration curve for 5-year survival

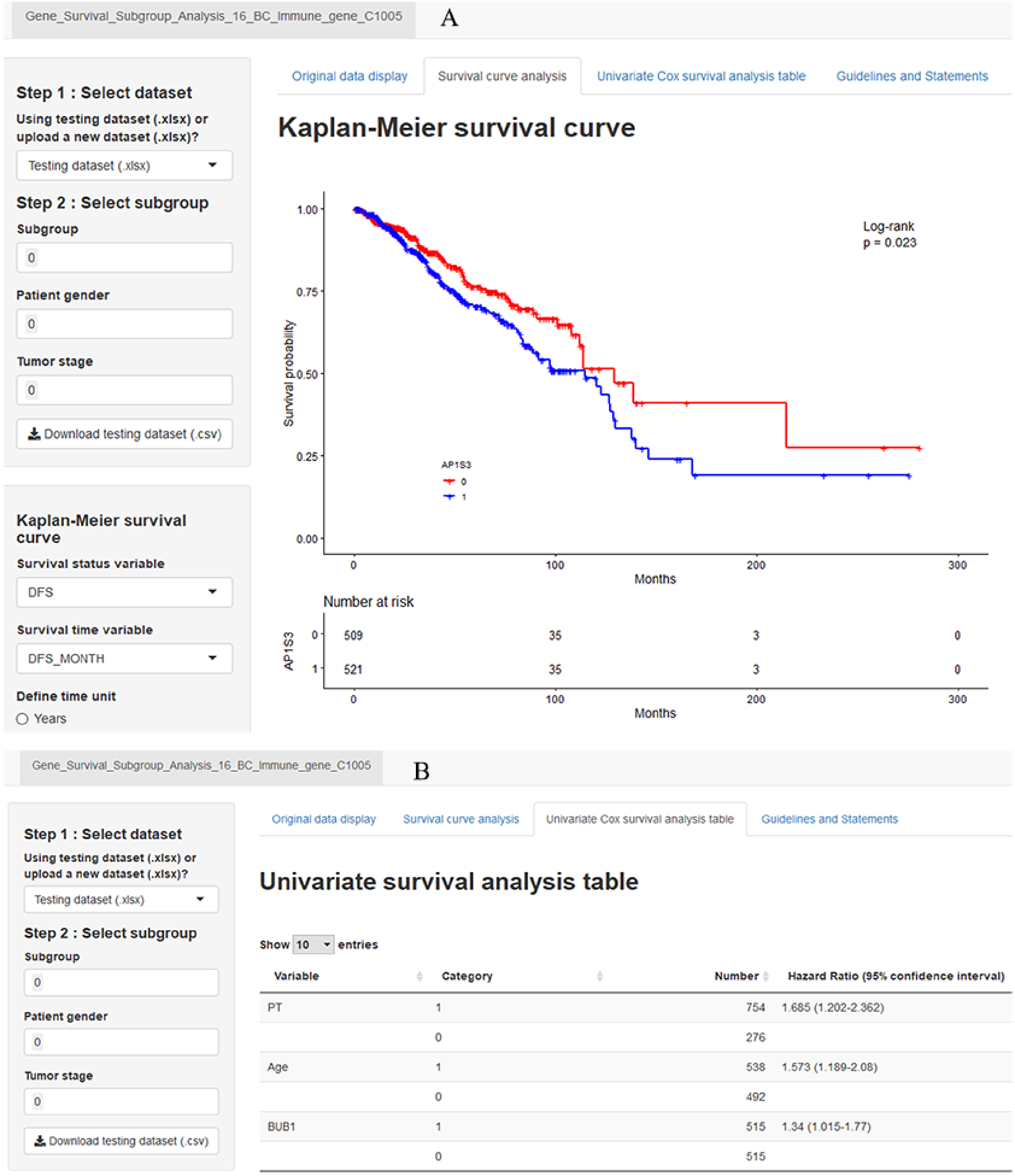

Supplementary Figure 8. Home page of Gene Survival Analysis Screen System: (A). Survival curves display page depicted and compared the survival curve between two defined subgroups; (B).Univariate survival analysis display page displayed results of univariate survival analysis for selected variables. Gene Survival Analysis Screen System allows users define different subgroups by themselves. Users are free to download, upload, and select the dataset for gene survival analysis.

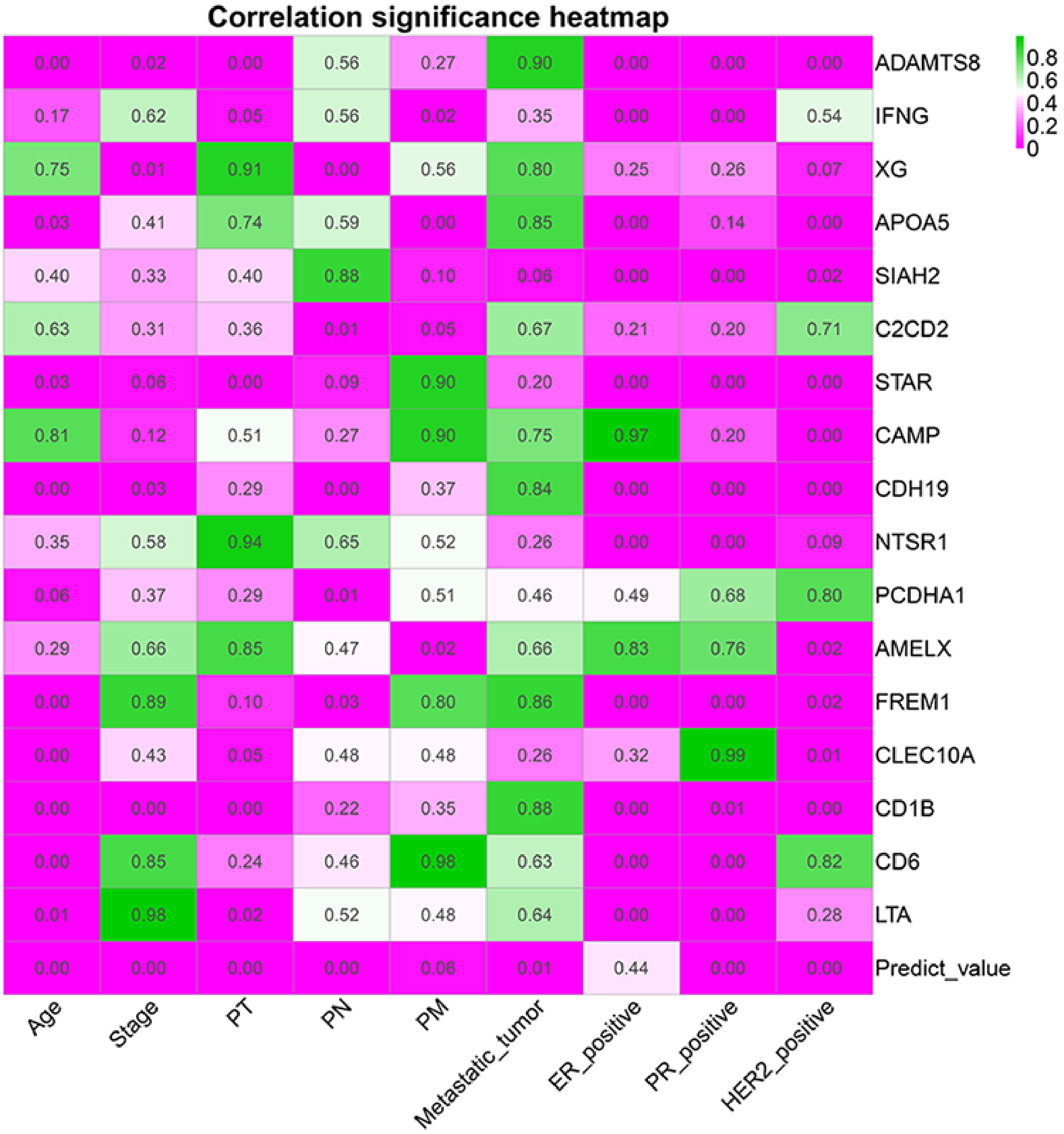

Supplementary Figure 9.Clinical variable correlation significance heatmap

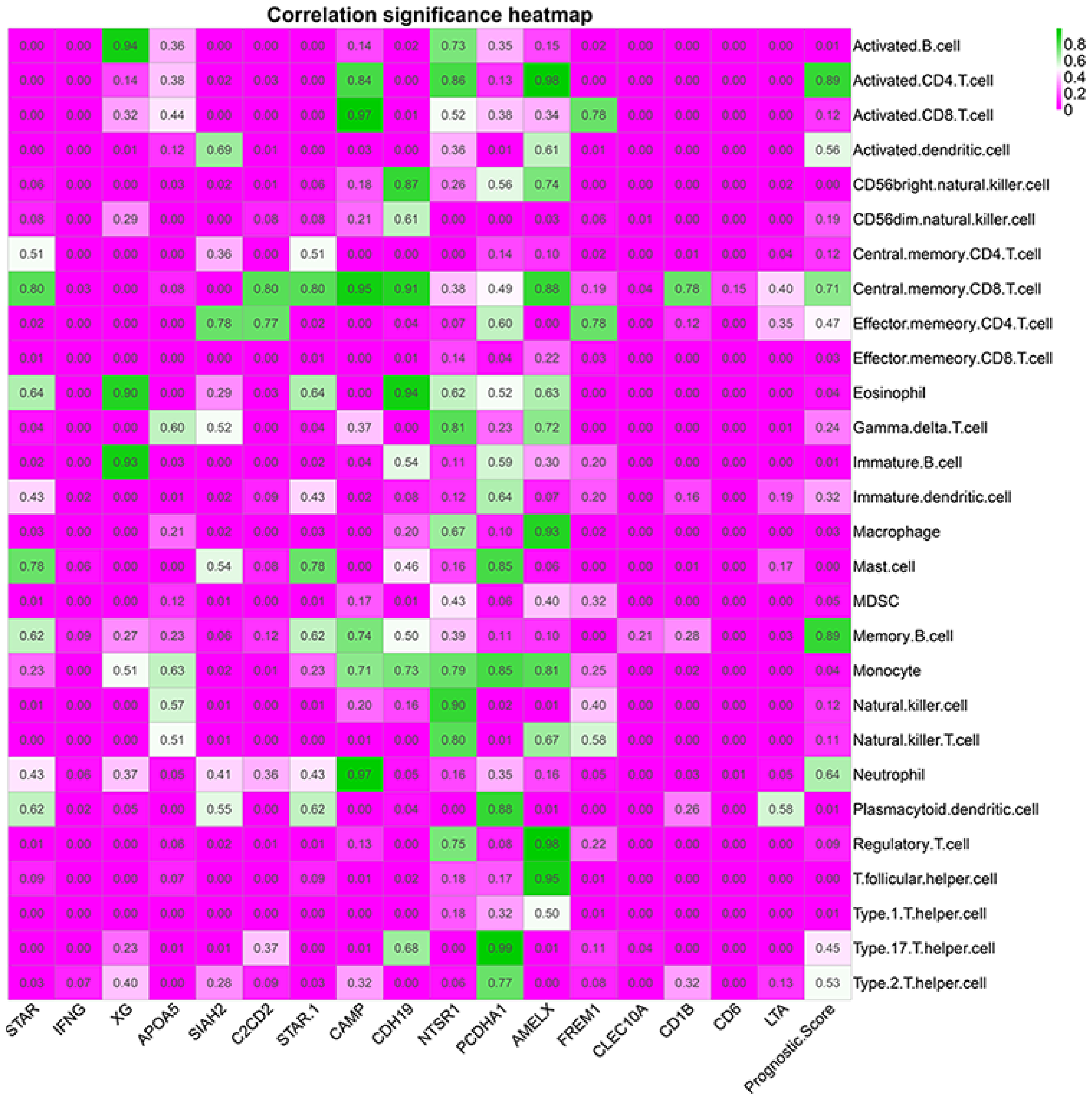

Supplementary Figure 10.Immune gene correlation significance heatmap

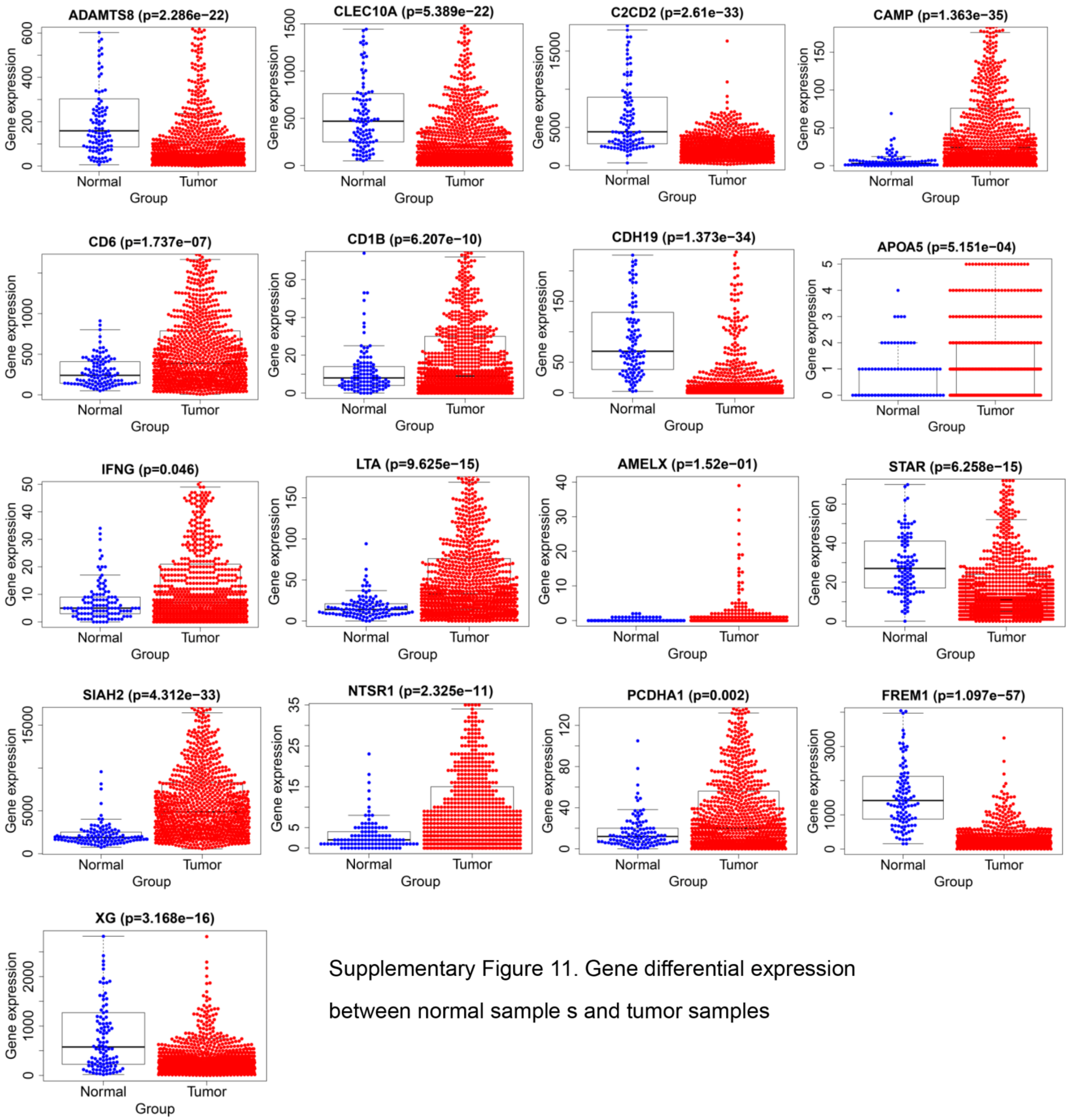

Supplementary Figure 11. Gene differential expression between normal sample s and tumor samples

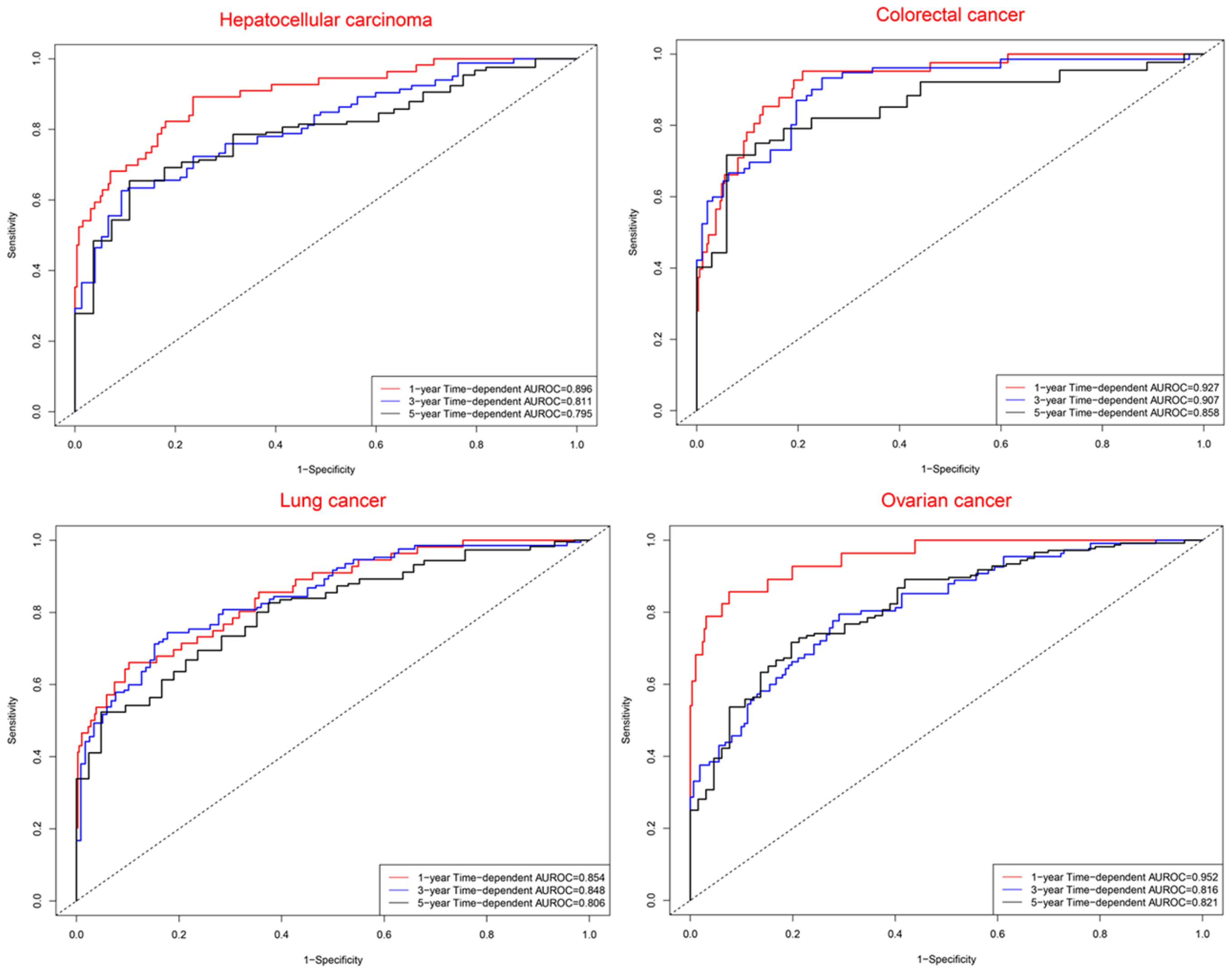

Supplementary Figure 12. Survival curves of four malignant solid tumors: (A). Hepatocellular carcinoma; (B). Colorectal cancer; (C). Lung cancer; (D). Ovarian cancer.

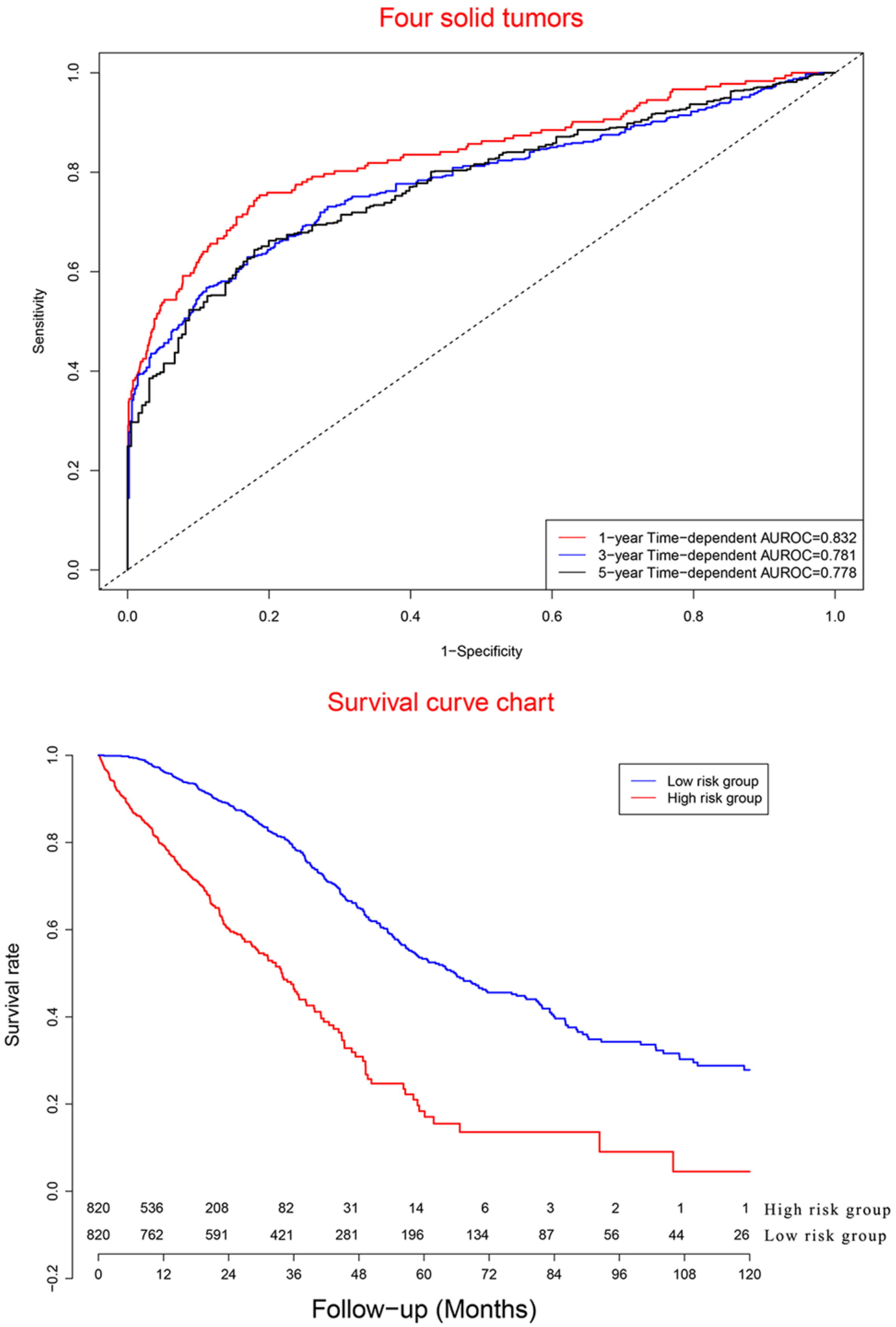

Supplementary Figure 13. Clinical performance in five malignant solid tumor datasets:
(A). Time-dependent receiver operating characteristic curves;
(B). Survival curves for high risk group and low risk group.

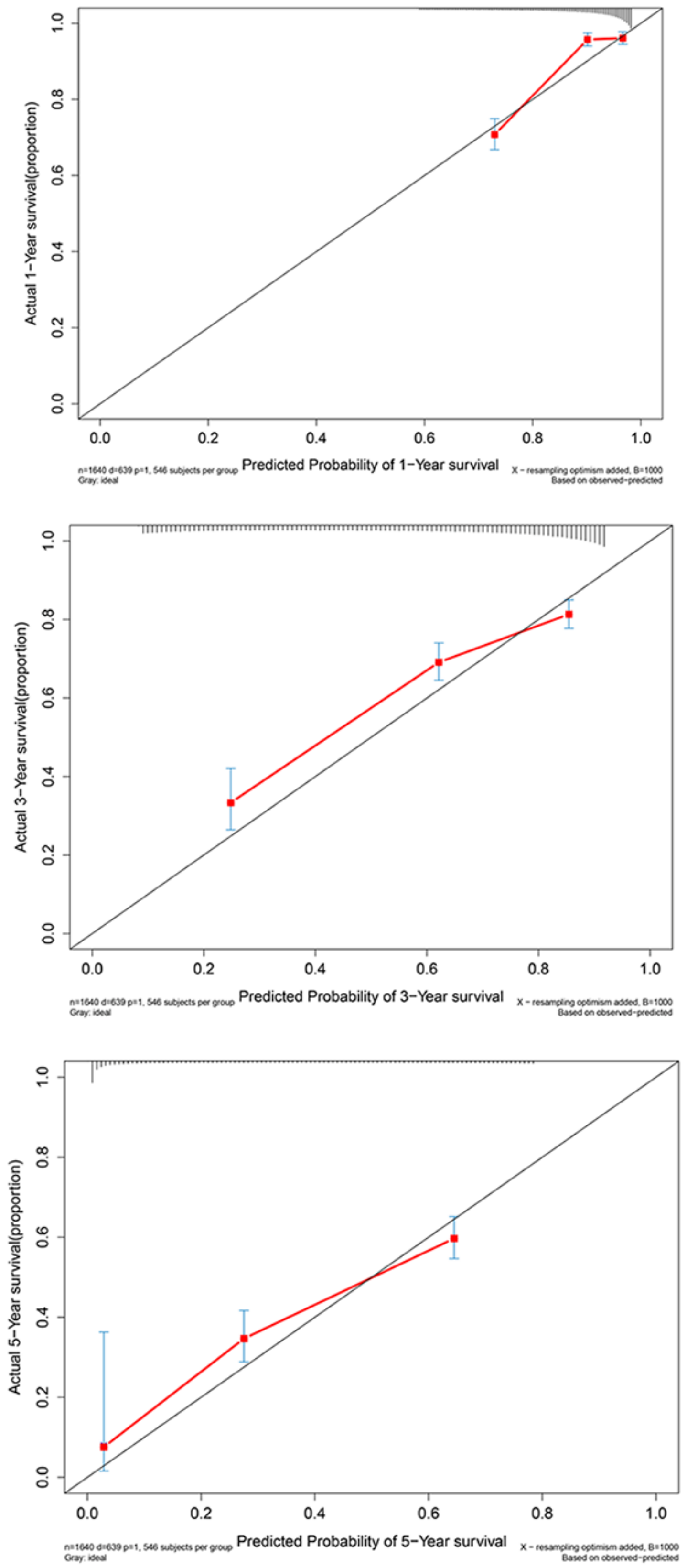

Supplementary Figure 14. Calibration curves in four malignant solid tumor datasets: (A). Calibration curve for 1-year survival; (B). Calibration curve for 3-year survival; (C). Calibration curve for 5-year survival
